# Impact of CD1d Deficiency on Metabolism

**DOI:** 10.1371/journal.pone.0025478

**Published:** 2011-09-29

**Authors:** Maya E. Kotas, Hui-Young Lee, Matthew P. Gillum, Charles Annicelli, Blas A. Guigni, Gerald I. Shulman, Ruslan Medzhitov

**Affiliations:** 1 Department of Immunobiology, Yale University School of Medicine, New Haven, Connecticut, United States of America; 2 Department of Internal Medicine, Yale University School of Medicine, New Haven, Connecticut, United States of America; 3 Department of Cellular and Molecular Physiology, Yale University School of Medicine, New Haven, Connecticut, United States of America; 4 Howard Hughes Medical Institute, Yale University School of Medicine, New Haven, Connecticut, United States of America; 5 Department of Neurology, University of Iowa, Iowa City, Iowa, United States of America; Louisiana State University, United States of America

## Abstract

Invariant natural killer T cells (iNKTs) are innate-like T cells that are highly concentrated in the liver and recognize lipids presented on the MHC-like molecule CD1d. Although capable of a myriad of responses, few essential functions have been described for iNKTs. Among the many cell types of the immune system implicated in metabolic control and disease, iNKTs seem ideally poised for such a role, yet little has been done to elucidate such a possible function. We hypothesized that lipid presentation by CD1d could report on metabolic status and engage iNKTs to regulate cellular lipid content through their various effector mechanisms. To test this hypothesis, we examined CD1d deficient mice in a variety of metabolically stressed paradigms including high fat feeding, choline-deficient feeding, fasting, and acute inflammation. CD1d deficiency led to a mild exacerbation of steatosis during high fat or choline-deficient feeding, accompanied by impaired hepatic glucose tolerance. Surprisingly, however, this phenotype was not observed in Jα18*^−/−^* mice, which are deficient in iNKTs but express CD1d. Thus, CD1d appears to modulate some metabolic functions through an iNKT-independent mechanism.

## Introduction

In recent years, it has become increasingly clear that molecules and cells classically associated with the immune system have important roles in maintenance of whole body energy metabolism. Several mouse strains deficient in cytokines or chemokines are either protected from or prone to obesity and insulin resistance [1]. In addition, nearly every cell type classically associated with the immune system—macrophages [2], [3], conventional T cells [4], [5], [6], eosinophils [7], mast cells [8], B cells [9]—has been implicated in the control or pathogenesis of obesity-associated morbidity. It has been suggested that inflammation may inhibit peripheral usage of glucose in order to spare energy for lymphocytes, whose activation depends directly on glucose availability [10]. However, any switch away from the normal homeostatic set point must be reversible; if energy redistribution is indeed a purposeful role of some components of the immune system, other components should turn back the switch when the immune response has resolved. An overarching rationale for how and why the various cells of the immune system orchestrate metabolic processes remains elusive.

Invariant natural killer T cells (iNKTs) are innate-like lymphocytes that co-express NK markers such as NK1.1 with a semi-invariant αβ T cell receptor and show an activated/memory phenotype even in naïve animals. Unlike conventional T cells, iNKTs are selected on and activated by the lipid-presenting MHC class 1b molecule CD1d, which is constitutively expressed on hepatocytes and other parenchymal cells, as well as antigen-presenting cells [11]. Also unlike conventional T cells, activation of iNKTs can occur through presentation of not only exogenous, pathogen-derived lipids but also endogenous lipids in combination with pro-inflammatory cytokines [12]. While present in typical lymphatic organs such as the spleen and bone marrow, iNKTs are surprisingly abundant in the liver, where they constitute up to 50% of liver lymphocytes [13]. Using the model lipid antigen α-Galactosylceramide to study iNKT biology, numerous effector functions have been described for these cells, including elaboration of a vast array of cytokines and chemokines, cytolytic activity, and activation of NK cells, conventional T cells, macrophages, B cells, and even regulatory T cells (Tregs) [11]. Yet the physiologic role of these cells remains ambiguous, largely because very few pathogen-derived CD1d ligands have been identified. Of note, while the major CD1d-restricted cell type is invariant NKTs, CD1d can also present lipids to other unconventional (NK)T cells, which are lesser in number and remain poorly understood [12].

The curious ability to sense lipids, along with the striking enrichment of iNKTs amongst hepatic lymphocytes led us and others to hypothesize that iNKTs might be involved in metabolic control. Several investigators have reported a pro-atherogenic role for iNKTs [14], [15], [16], [17], [18], and others have observed that the proportion of hepatic iNKTs is selectively reduced in obese animals [19], [20], [21]. Consistent with a pathological role of iNKTs in obesity and insulin resistance, administration of the NKT activator α-Galactosylceramide has been reported to worsen insulin resistance in obese mice [22]. However, another study reported that adoptive transfer of NKTs improved steatohepatitis and glucose intolerance [23], supporting a beneficial role for these cells. For these reasons and because iNKTs can engage a wide array of effector mechanisms, we felt they were well poised to participate in metabolic regulation, but that such a role had been understudied.

Like iNKTs, CD1d itself has several characteristics that suggest a role in metabolism. According to public databases, its expression is highest in liver, white adipose and brown adipose tissue—all important metabolic organs—rather than on professional antigen-presenting cells. And although constitutively expressed on many cell types, CD1d expression is further inducible by PPARγ [24], a master regulator of lipid metabolism that is activated by fatty acids and other lipids. Thus, much like pathogen-associated molecules promote MHC class II antigen processing and presentation, lipid ligand availability may increase CD1d expression. Finally, CD1d shares trafficking and lipid-loading machinery with lipoprotein metabolism, where ligands are delivered using lipoprotein machinery and loaded onto CD1d using the VLDL lipidator, microsomal triglyceride transfer protein (MTP) [12], [25]. Thus, we hypothesized that, like other classical and non-classical MHC molecules [26], CD1d reports on cellular status and stress. During states of lipid imbalance or overload, CD1d may alert iNKT cells, allowing the immune system to modify the lipid content of hepatocytes and other metabolically important cells.

To test this hypothesis, we presented CD1d deficient (*CD1d^−/−^*) mice—which lack CD1d-restricted T cells due to failure of positive thymic selection [27]—with various metabolic stresses including high fat feeding, choline-deficient diet, starvation, and endotoxin injection. In the former but not the later two models, we found that *CD1d^−/−^* mice had a subtle but consistent increase in hepatic triglyceride (TG) accumulation relative to wild type, possibly due to an increased hepatic lipid uptake from circulation. This was coupled with a slight worsening of glucose intolerance that was likely attributable to defective hepatic insulin response. In every other respect studied, however, *CD1d^−/−^* mice were indistinguishable from wild type controls using available methodology. Interestingly, in the same high fat feeding model, we observed no difference between *Jα18^−/−^* and wild type mice, suggesting that the slight worsening of metabolic function in *CD1d^−/−^* mice is due to non-invariant NKTs, or is attributable for a T cell-independent function of CD1d.

## Materials and Methods

### Animal Care and Maintenance

*CD1d^−/−^* mice (deficient in both *CD1d1* and *CD1d2*) on a B6 background (backcrossed ≥10 times), *Jα18^−/−^* mice on a B6 background (backcrossed ≥6 times), C57Bl6 mice (NCI or Jackson Laboratories), and ob/ob mice (6–10 weeks old, Jackson Laboratories) were maintained on a constant 12-h light:12-h dark cycle with free access to water and *ad libitum* access to standard chow diet (2018s, Harlan Teklad, Madison, WI) unless otherwise specified. For metabolic phenotyping, male *CD1d+/−* mice were bred to generate knockouts and littermate controls, although results did not differ from those performed with B6 mice purchase from NCI or Jackson. Mice were maintained on standard chow until 6–8 weeks of age before switching to high fat diet (60% kCal from fat, D12492, Research Diets, New Brunswick, NJ) for 8–16 weeks (depending on the experiment) or choline-deficient diet (TD.88052, or TD.03118 for controls, Harlan Teklad) for 4 weeks. Age matched controls were used for all experiments with altered diets. Chow-fed mice were studied at 7–10 weeks of age. For fasting experiments, *ad libitum* chow-fed mice (8–10 weeks old) were deprived of food with free access to water starting shortly after the start of the light cycle and blood was collected at the indicated intervals. For LPS injection, *ad libitum* fed mice were injected with 100 ug LPS i.p., and deprived of food for the next 6 hours, after which livers were harvested and flash frozen for RNA preparation. This study was carried out in accordance with the recommendations in the Guide for the Care and Use of Laboratory Animals of the National Institutes of Health. All procedures were approved by the Institutional Animal Care and Use Committee (IACUC) of Yale University (Protocol #2008-08006).

### Metabolic parameters

Metabolic rate, food intake and activity were measured using the Comprehensive Laboratory Animal Monitoring System (Columbus, OH) over 48 hours, and body composition by *in vivo* ^1^H magnetic resonance spectroscopy (MiniSpec, Bruker). For lipid analyses, plasma harvested from overnight fasted or fasted/refed (4 hrs) were snap frozen in liquid nitrogen and stored at −80C until analysis. Triglyceride and NEFA were measured using enzymatic kits (Triglyceride SL, Genzyme Diagnostics, Canada and NEFA-HR Wako, Richmond, VA) according to the manufacturer's instructions. For tissue lipid analysis, tissues were collected from overnight fasted mice (unless otherwise noted), snap frozen, and stored at −80 until analysis. Lipids were extracted by 2∶1 chloroform∶methanol according to the Folsch method [28] from equal masses of tissue and then assayed using enzymatic kits (Triglyceride SL and Cholesterol Assay Kit, Cayman Chemical, Ann Arbor, MI) or by ^31^P NMR (Avanti, Alabaster, AL). To measure hepatic lipid export, 4 hr-fasted mice were injected with 1 g/kg poloxamer 407 (Pluronic F-127, Sigma) and plasma was collected at the indicated timepoints for analysis by Triglyceride SL. For microscopy, mice were fasted overnight prior to sacrifice. Livers were fixed in 4% paraformaldehyde for 8 hours, subjected to a sucrose gradient to preserve architecture, and embedded in OCT freezing media. Frozen sections were stained with Oil Red O as described [29] and visualized by light microscopy (Nikon).

### Glucose Tolerance Tests (GTT), Insulin Tolerance Tests (ITT), and Pyruvate Tolerance Tests (PTT)

For the GTT, overnight fasted mice were injected i.p. with 1.0 g/kg (for *CD1d^−/−^* and WT) or 0.75 g/kg (for *Jα18^−/−^* and WT) glucose. Blood was sampled from the retro-orbital plexus before injection and at the indicated times and glucose was measured using a OneTouch Ultra glucometer (LifeScan, Milpitas, CA). Concurrently collected plasma samples were frozen at −20C until insulin ELISA (Crystal Chem). For the ITT, 4 hr-fasted animals were injected i.p. with 1.0 U/kg of human recombinant insulin (Novolin, Novo Nordisk, Denmark). For the PTT, overnight fasted mice were injected i.p. with 2.0 g/kg sodium pyruvate.

### Hyperinsulinemic-Euglycemic Clamp

A jugular venous catheter was implanted 6 to 7 d before the hyperinsulinemic-euglycemic clamps. To assess basal whole-body glucose turnover, after overnight fast, [3-^3^H]-glucose (HPLC purified; Perkin-Elmer Life Sciences) was infused at a rate of 0.05 µCi/min for 120 min into the jugular catheter. Following the basal period, hyperinsulinemic-euglycemic clamps were conducted in conscious mice for 140 min with a 3 min primed (31.5 mU/kg) followed by a continuous [4.5 mU/(kg-min)] infusion of human insulin (Novolin; Novo Nordisk), a continuous infusion of [3-^3^H]-glucose (0.1 µCi/min), and a variable infusion of 20% dextrose to maintain euglycemia (∼120 mg/dL). Plasma samples were obtained from the tip of the tail at 0, 30, 50, 65, 80, 90, 100, 110, 120, 130 and 140 min. The tail incision was made at least 2 h before the first blood sample was taken to allow for acclimatization, according to standard operating procedures [30]. Also, mice received an i.v. albumin-containing solution mimicking artificial plasma during the insulin-stimulated period of the clamp to compensate for volume loss secondary to blood sampling. At the end of the clamps, mice were anesthetized with pentobarbital sodium i.v. injection (150 mg/kg) and all tissues were taken within 4 min, snap-frozen in liquid nitrogen, and stored at −80°C for subsequent analysis. Plasma glucose (10 µL per sample) was measured using a YSI 2700D glucose analyzer. For the determination of ^3^H-glucose, plasma was deproteinized with ZnSO_4_ and Ba(OH)_2_, dried to remove ^3^H_2_O, resuspended in water, and counted in scintillation fluid (Ultima Gold; Perkin-Elmer Life Sciences).

Rates of basal and insulin-stimulated whole-body glucose turnover were determined as the ratio of the [3-^3^H]-glucose infusion rate (disintegrations per minute, dpm) to the specific activity of plasma glucose (dpm/mg) at the end of the basal period and during the final 30 min of steady state of the clamp, respectively. Endogenous glucose production was calculated by subtracting the glucose infusion rate from the whole-body insulin-stimulated glucose disposal. Whole body glycolysis rate was estimated by the ratio of delta ^3^H_2_O [dpm/(mL-min)] to the specific activity of plasma glucose (dpm/mg) at the end of the basal period and during the final 30 min of the clamp.

### Flow Cytometry

iNKTs were analyzed using a variation of published methods [31]. In brief, spleens were mechanically dissociated using frosted glass slides and red blood cells were removed with Ack lysis buffer (Gibco). Livers were mechanically dissociated and mononuclear cells were isolated by centrifugation on a 67.5%/44% isotonic Percoll gradient (GE Healthcare). Bilateral epididymal white adipose tissue (WAT) was dissociated in PBS containing Liberase TM (Roche) and stromal vascular cells (pellet) were separated from adipocytes (floating) by centrifugation. Cell surface staining was performed using commercially available antibodies against B220 (clone RA3-6B2), CD3ε (145-2C11), CD4 (GK1.5), CD8α (53-6.7), CD69 (H1.2F3), CD25 (PC61.5), NK1.1 (PK136) and BrdU (BD Biosciences and eBioscience), and Annexin V (Invitrogen). PBS57-loaded CD1d tetramers and empty tetramer were provided by the NIH tetramer facility (Emory University, Atlanta, Georgia) and regularly titrated to optimal concentration. For BrdU incorporation experiments, 6-month high fat fed mice were injected i.p. daily with 1 mg BrdU for 7 days. Cells were analyzed using a BrdU flow cytometry kit (BD Biosciences). Cells were analyzed on FACSCalibur or LSR II (BD Biosciences) using FACSDiva software (BD Biosciences) and FlowJo (Treestar).

### Quantitative reverse transcription-polymerase chain reaction (qRT-PCR)

For analysis of gene expression, total RNA was isolated from liver, hypothalamus, or epididymal white adipose tissue by phenol/chloroform extraction followed by cleanup with RNeasy (Qiagen) according to the manufacturer's instructions. Hypothalami were excised from the brain along the borders of the anterior commisure and third ventricle. Poly(A) mRNA was reverse transcribed and PCR was performed using intron-spanning gene specific primers (sequences are available upon request) and SYBR green master mix (Qiagen or Quanta) on a Stratagene machine (Agilent Technologies). Fold change in mRNA expression was determined using the ΔΔcT method normalize to *HPRT*, *RPL13a*, or both. A list of examined genes is included in Table S1.

### Protein analysis

CXCL16 expression was measured using a RayBio Cytokine Array III membrane according to the manufacturer's instructions. Spot density was determined using Image J and normalized to positive control spots.

### Statistical Analysis

Data are expressed as means ± SEM. Statistical significance was determined by t-test or two-way ANOVA, as appropriate. Statistically significant results are reported for p<0.05.

## Results

### iNKTs are selectively decreased in obese livers

**Figure 1:**
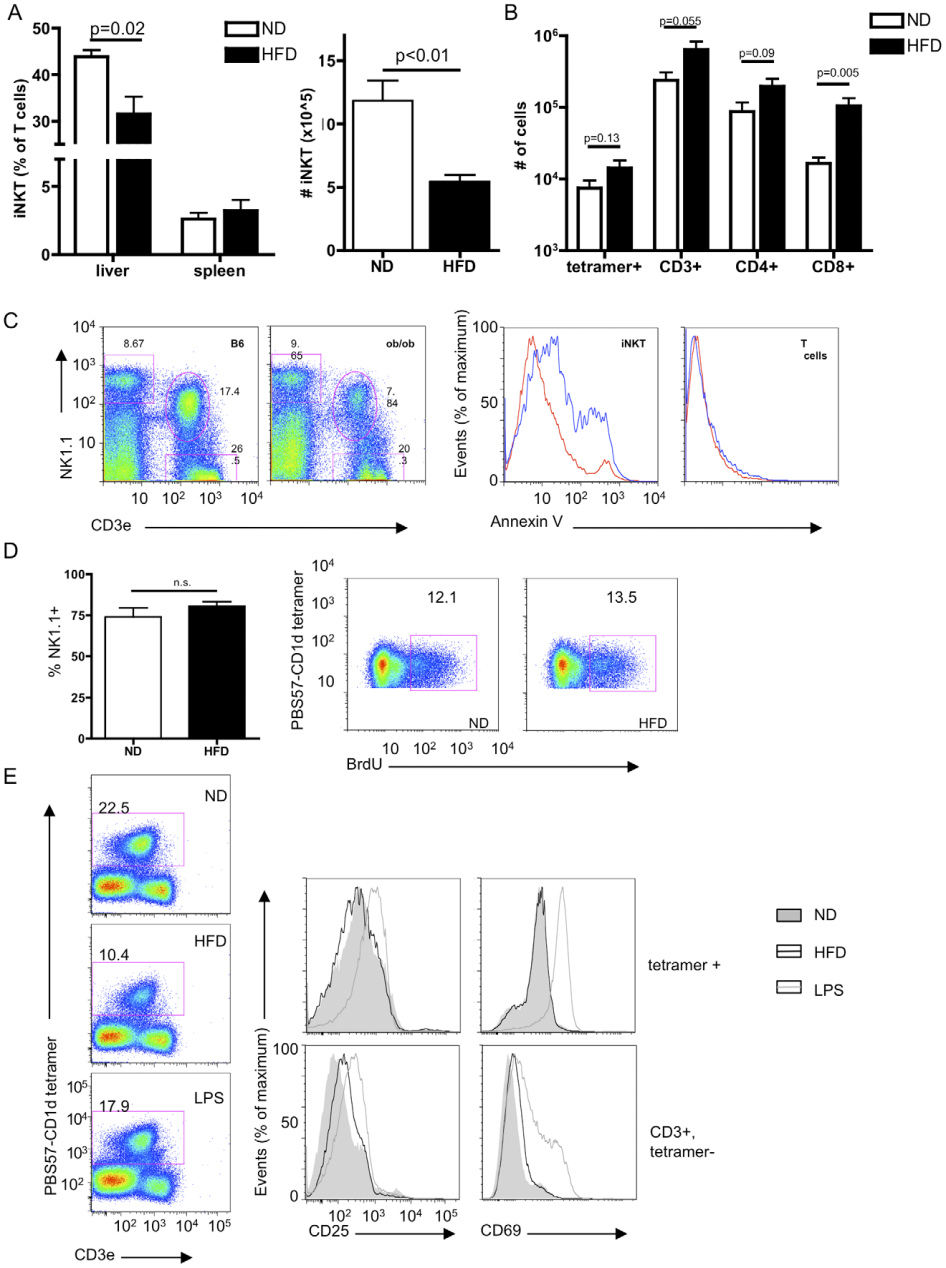
iNKTs are selectively decreased in obese livers. A) The percentage (left panel) of tetramer+, CD3ε-int iNKTs among total CD3ε+B220- lymphocytes is decreased in the liver but not the spleen of mice made obese through 6 months of HFD compared to controls, as is the absolute number of hepatic iNKTs (right panel) (n = 4 mice/group). B) The number of CD8+ T cells in the epididymal white adipose tissue of 4 month HFD-fed mice is increased relative to controls, while the numbers of tetramer+ iNKTs, total CD3ε+, tetramer- non-iNKTs, and CD3ε+, tetramer-, CD4+ cells is non-significantly increased (n = 11/group). C) ob/ob mice (blue line) have an increased percentage of AnnexinV+ cells among NK1.1+, CD3ε+ hepatic iNKTs but not conventional T cells, compared with lean controls (red line). Representative of 2 experiments. D) The percentage of (left panel) NK1.1+ hepatic iNKTs (gated on B220-, CD3ε-int, tetramer+ cells) does not differ between chow-fed and 4 week HFD-fed mice (n = 4/group), nor does BrdU incorporation into iNKTs (representative of 2/group). E) Hepatic iNKTs from 9-week HFD-fed mice do not show evidence of acute activation (representative of 3 experiments).

Consistent with previous reports [19], [20], [21], [32], we found that high fat feeding led to a significant reduction of PBS57-CD1d tetramer+ iNKTs in the liver (Figure 1A&E) but not in the spleen (Figure 1A). However, when expressed as an absolute number of cells but not as a percentage, iNKTs were slightly increased in WAT. This difference between percentage and numbers of iNKTs in the WAT was attributable to the greater number of WAT stromal vascular cells isolated from obese animals, such that an increase in WAT iNKT numbers was dwarfed by much larger increases in other infiltrating populations; this is consistent with the dramatic increase of macrophages and other inflammatory cells observed during obesity [3]. However, when expressed as an absolute number of cells, iNKTs, conventional T cells and CD4+ T cells were slightly increased, and CD8+ cells were dramatically increased (Figure 1B).

Depletion of iNKTs from obese livers could be due to at least one of four potential causes: increased apoptosis, defective homeostatic maintenance, activation-induced death or receptor internalization, or migration elsewhere. We observed an increased percentage of Annexin V positive cells among NK1.1+CD3ε+ cells in obese mouse livers (Figure 1C), consistent with published results in high fat-fed mice [20]. However, we sought to address the possibility that other factors were also contributing to a reduced iNKT population. Recent thymic emigrant iNKTs are largely NK1.1-, and acquire NK1.1 in the periphery in the CD1d-dependent manner [33]. We observed no difference in NK1.1 staining, or in BrdU incorporation into hepatic iNKTs (Figure 1D), suggesting that neither proliferation nor maturation of iNKTs were altered in the obese state. We did not observe any increase in staining for activation markers such as CD69 or CD25 in hepatic iNKTs from obese mice, although, interestingly, we observed a small shift in these markers in hepatic CD3ε+, tetramer- T cells (Figure 1E). We also did not observe increased PD-1 staining in hepatic iNKTs from obese mice, although this has been determined to be a marker of chronically activated and/or exhausted iNKTs [34], [35]. These data suggest that iNKTs are not activated by chronic high fat diet or in the obese state, although we cannot rule out the possibility that activation may have occurred at some earlier time.

### CD1d and CXCL16 expression are changed by metabolic state

**Figure 2:**
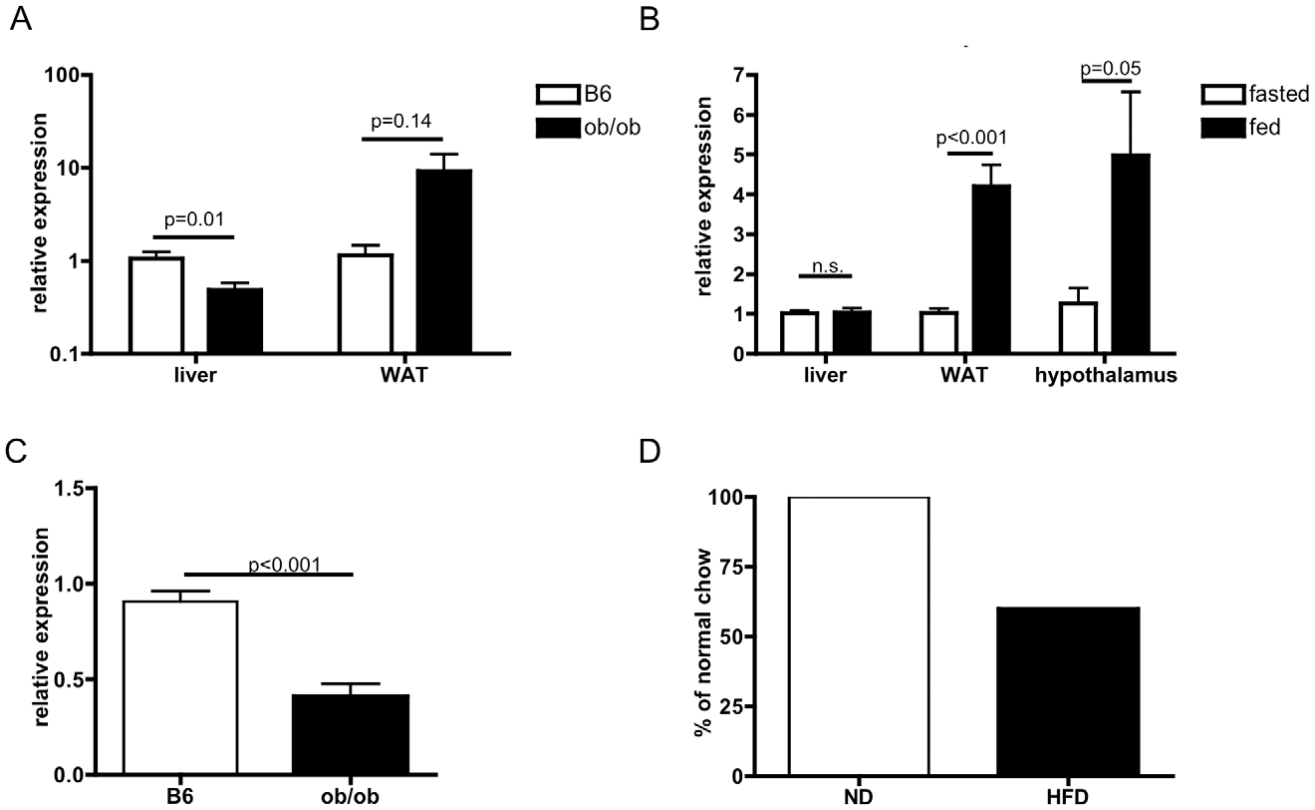
*CD1d* and *CXCL16* expression are changed by metabolic state. A) *CD1d* mRNA expression is reduced in livers of ob/ob mice, whereas it is non-significantly increased in the WAT (n≥5/group). B) *CD1d* mRNA expression is induced by feeding in the hypothalamus and WAT, but not the liver, of lean B6 mice (n≥4/group). C) *CXCL16* mRNA expression is reduced in the livers of ob/ob mice (n = 5–6/group). D) CXCL16 protein level in mice fed regular chow or high fat diet for 3 months (pooled from 5/group).

If iNKTs regulate metabolism, it would be expected that CD1d presentation or iNKT cell localization would be altered by changes in metabolic state. We found that *CD1d* mRNA expression was significantly reduced in livers, and trended to increase in WAT of genetically obese ob/ob mice (Figure 2A). Interestingly, *CD1d* expression in the WAT and hypothalamus was also sensitive to acute changes in feeding and fasting in lean animals, although expression was not altered in the liver under these conditions (Figure 2B). CXCL16∶CXCR6 interactions are thought to contribute to iNKT homing to and retention in the liver [36], [37]. We found that *CXCL16* mRNA and protein expression was dramatically reduced in obese mice relative to lean controls (Figure 2C&D), offering an alternative explanation for the reduced hepatic iNKT numbers seen in the obese state.

### *CD1d^−/−^* mice are metabolically normal when fed a standard diet, and gain weight equally on a high fat diet

**Figure 3:**
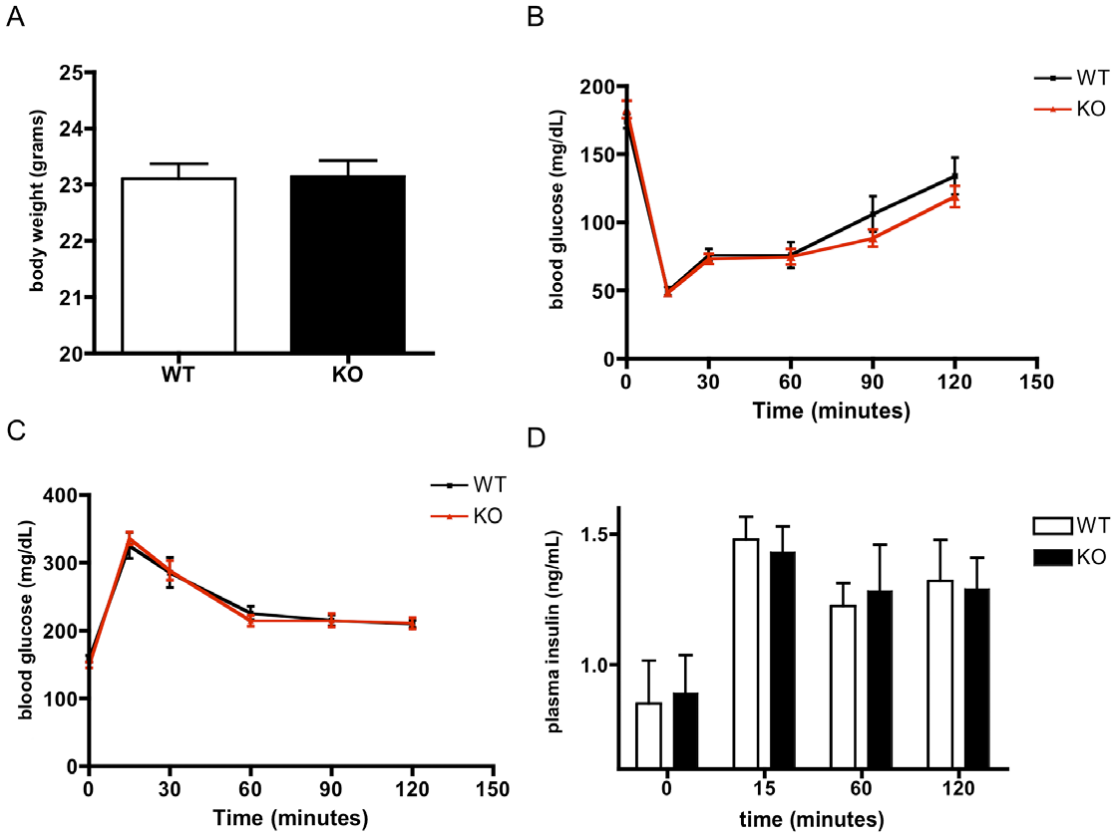
*CD1d^−/−^* mice are metabolically normal when fed a standard chow diet. A) *CD1d^−/−^* mice and their littermates have similar body weight on normal chow (n = 11–12/group). B) Chow-fed *CD1d^−/−^* and WT mice have identical insulin tolerance tests (n = 9–12/group) and C) glucose tolerance tests. D) Plasma insulin does not differ during a glucose tolerance test (n = 9–12/group).

To address whether alterations in liver CD1d expression and/or iNKTs reflected a cause or consequence of metabolic changes during obesity, we examined the physiology of lean, chow-fed *CD1d^−/−^* and controls. We found that the body weight of these animals was indistinguishable (Figure 3A), and that the plasma glucose response to an insulin tolerance test (Figure 3B) or glucose tolerance test (Figure 3C) was identical. Furthermore, insulin secretion in response to a glucose tolerance test did not differ (Figure 3D). Thus *CD1d^−/−^* mice appear normal on a standard diet.

### CD1d deficiency worsens DIO-associated glucose intolerance

**Figure 4:**
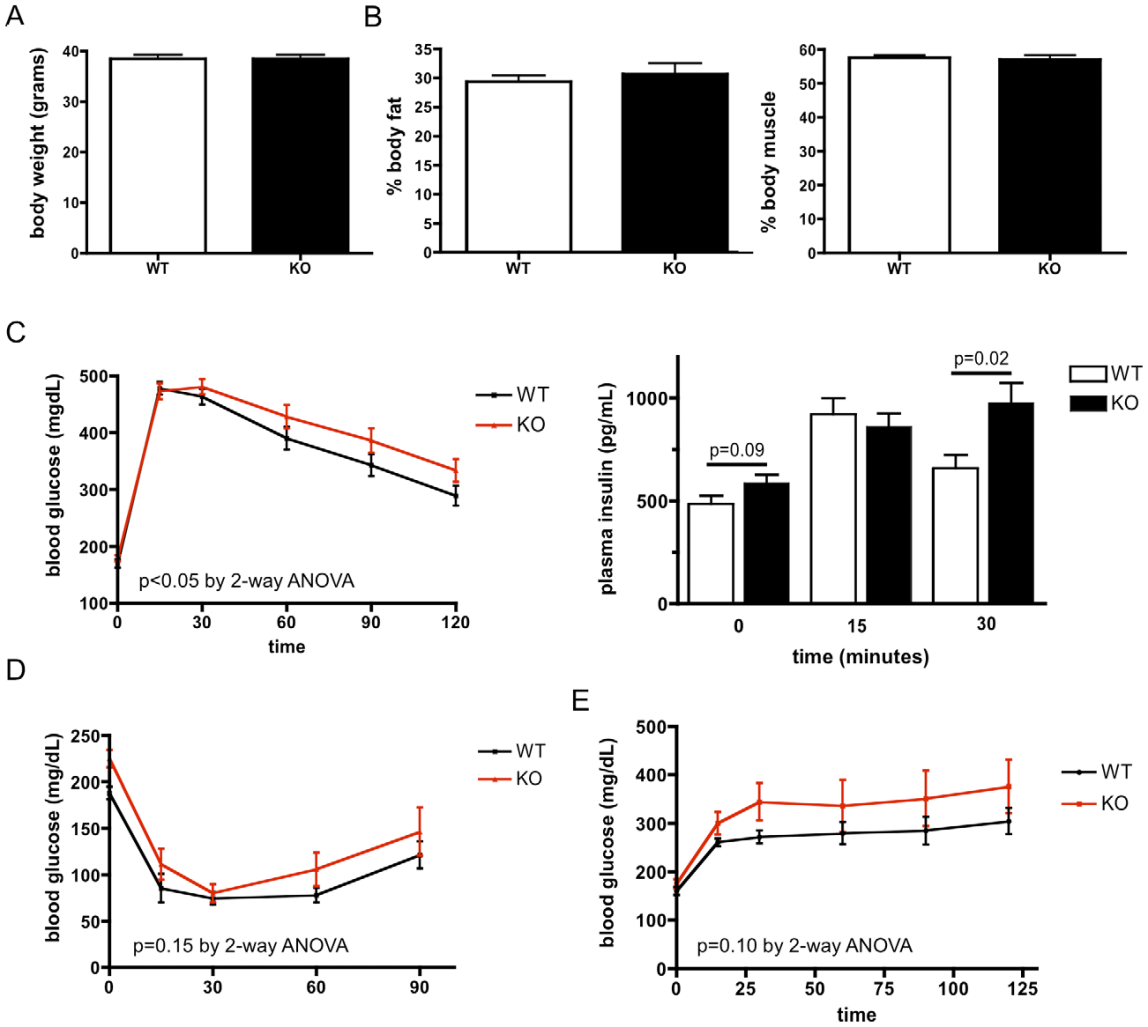
CD1d deficiency worsens DIO-associated glucose intolerance. A) Body weight and B) composition do not differ between high fat-fed *CD1d^−/−^* and littermates (n = 8/group; representative of 2 experiments). C) Glucose tolerance tests demonstrate a slight but statistically significant worsening of glucose intolerance (left panel) in high fat-fed *CD1d^−/−^* mice (n = 15–16/group). Plasma insulin (right panel) during a glucose tolerance test is higher in *CD1d^−/−^* mice. D) *CD1d^−/−^* mice show a strong trend towards reduced response during an insulin tolerance test (n = 10–11/group). E) *CD1d^−/−^* mice show a strong trend towards increased blood glucose during a pyruvate tolerance test (n = 5–6/group).

To probe the role of CD1d in diet-induced obesity (DIO), *CD1d^−/−^* mice were fed a high fat diet (60% kCal from fat for 12 weeks). *CD1d^−/−^* mice gained the same amount of weight as controls (Figure 4A) and had indistinguishable fat and muscle composition (Figure 4B). Caloric intake, metabolic rate, activity, and whole body substrate utilization were not different between genotypes (Table 1). However, when challenged with an intraperitoneal glucose bolus, we found that obese *CD1d^−/−^* mice demonstrated slightly worsened glucose intolerance compared to controls (Figure 4C, left panel). Of note, by performing at least 7 similar experiments, we determined that a minimum of ∼12 animals per group were required to reliably observe a statistically significant difference; this demonstrates that the effect size observed is quite small. Fasting plasma glucose and insulin measured at the start of glucose tolerance testing (GTT) did not differ between genotypes (Figure 4C), although there was a strong trend for increased fasting plasma insulin concentrations in *CD1d^−/−^* mice, consistent with a trend to increased insulin resistance. Plasma insulin excursion during the GTT did not significantly differ between groups (p = 0.19 by 2 way ANOVA), although *CD1d^−/−^* mice demonstrated increased plasma insulin concentrations at 30 minutes, past the peak of insulin secretion in control mice (Figure 4C, right panel). These data indicate that worsened insulin resistance, rather than defective insulin secretion, is the most likely cause of worsened glucose intolerance in *CD1d^−/−^*mice. An insulin tolerance test (ITT, Figure 4D) and pyruvate tolerance test (PTT, Figure 4E) demonstrated a consistently strong trend to reduced insulin sensitivity in *CD1d^−/−^* mice, although these tests did not reach statistical significance. Thus, CD1d plays a subtle role in regulating normal glucose and insulin metabolism during DIO.

**Table 1:** High fat-fed *CD1d^−/−^* mice do not differ from littermates in food intake, energy expenditure or activity.

	WT	KO	t-test
**VO2 (ml/kg/min)**	2432.53+/−40.79	2412.53+/−60.74	0.79
**VCO2 (ml/kg/min)**	1886.74+/−39.25	1874.72+/−40.55	0.83
**RER**	0.78+/−0.01	0.78+/−0.00	0.81
**heat (kcal/kg/hr)**	11.60+/−0.21	11.48+/−0.28	0.72
**feeding (g/kg/hr)**	2.48+/−0.14	2.51+/−0.07	0.84
**activity (counts/hr)**	54.22+/−4.79	60.30+/−6.97	0.48

Comprehensive Laboratory Animal Monitoring System data demonstrates no difference in VO2, VCO2, RER, heat, feeding, or activity between obese *CD1d^−/−^* mice and their littermates (n = 8/group).

### Glucose intolerance in *CD1d^−/−^* mice is attributable to decreased hepatic insulin sensitivity

**Figure 5:**
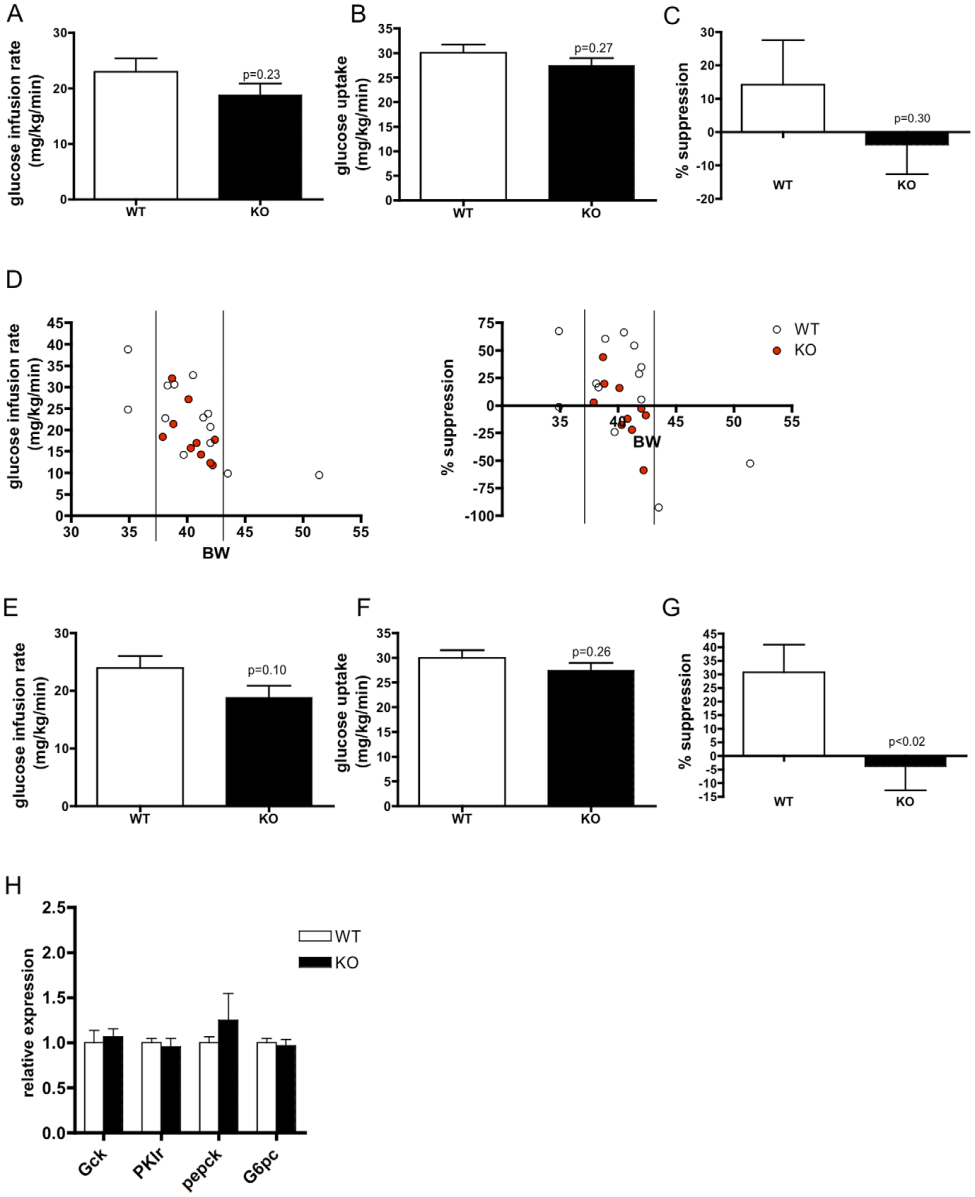
Glucose intolerance in *CD1d^−/−^* mice is attributable to decreased hepatic insulin sensitivity. A) Glucose infusion rate, B) peripheral glucose uptake, and C) suppression of hepatic glucose output suggest a trend towards hepatic insulin resistance in high fat-fed *CD1d^−/−^* mice (n = 9–13/group). D) Body weight strongly predicts decreased glucose infusion rate and poor suppression of hepatic glucose output; body weight matched group is shown within the vertical lines. E) Glucose infusion rate, F) peripheral glucose uptake, and G) suppression of hepatic glucose output excluding body weight outliers shows a significant defect in hepatic insulin sensitivity in *CD1d^−/−^* mice (n = 9–10/group). H) Gene expression from livers of WT and *CD1d^−/−^* mice (n = 8/group).

To explore the mechanism of exacerbated glucose intolerance in *CD1d^−/−^* mice, we performed hyperinsulinemic-euglycemic clamp studies [30]. We observed trends towards reduced insulin-stimulated whole body glucose metabolism (Figure 5A), reduced peripheral glucose uptake (Figure 5B) and reduced suppression of hepatic glucose output (Figure 5C). Body mass is a strong predictor of insulin insensitivity, (Figure 5D), with animals of increasing body weight generally showing reduced whole body insulin sensitivity and poorer suppression of hepatic glucose production; thus, it is recommended that tests of glucose homeostasis compare weight-matched mice [30]. Because our cohort of wild-type animals had a large variance in body weight, which could introduce substantial variation in measurement and obscure genotype-dependent differences, we reanalyzed our data using a weight-matched subset that excluded statistical or body weight outliers. These data demonstrated a strong trend to reduced whole body insulin sensitivity (Figure 5E) that was attributable to a significantly impaired suppression of hepatic glucose output (Figure 5G), rather than a defect in peripheral glucose uptake (Figure 5F). This suggested that gluconeogenic output and/or glycogenolysis may be increased in high fat-fed *CD1d^−/−^* mice relative to controls. To explore this mechanism, we performed quantitative RT-PCR on livers, but did not observe any differences in mRNA expression of glycolytic (*glucokinase*, *Gck* or *pyruvate kinase*, *PKlr*) or gluconeogenic (*phosphoenol pyruvate carboxykinase*, *pepck* or *glucose-6-phosphatase*, *G6pc*) genes in the fasted state (Figure 5H). It is possible, however, that gene expression may be abnormal in the hyperinsulinemic state, since basal endogenous glucose production can be normal in states of insulin resistance [38].

### CD1d deficiency worsens hepatic steatosis on a high fat diet

**Figure 6:**
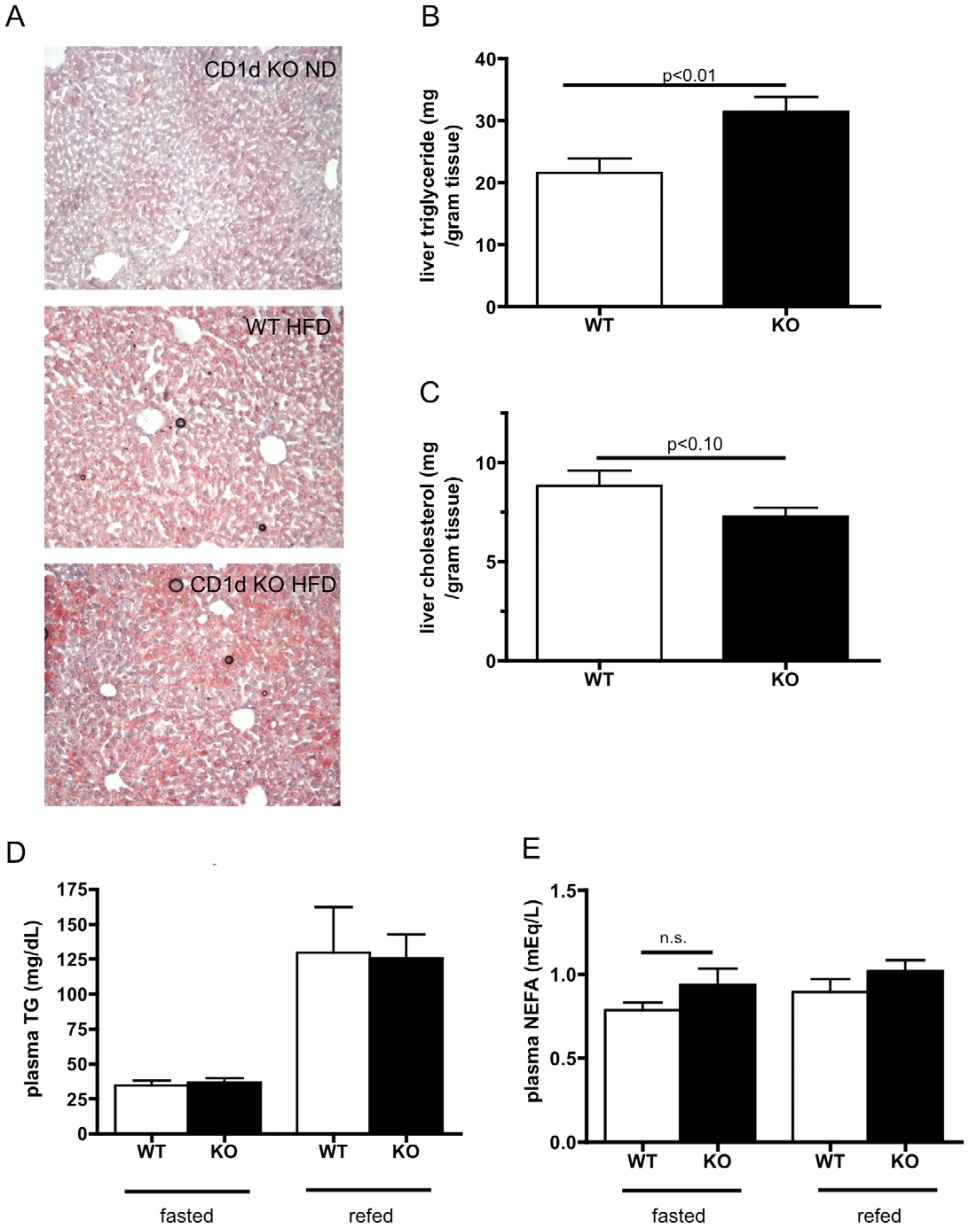
CD1d deficiency worsens hepatic steatosis on high fat diet. A) Representative oil red O staining shows that *CD1d^−/−^* mice are not steatotic on a normal diet, but develop more severe hepatic steatosis on a high fat diet. B) Quantitative analysis shows *CD1d^−/−^* mice have significantly more hepatic triglyceride accumulation than WT on a high fat diet (n = 12–14/group). C) Hepatic cholesterol content trends lower in *CD1d^−/−^* mice (n = 8/group). D) Plasma triglyceride does not differ in either the fasted or refed state (n = 5–6/group). E) Plasma free fatty acids do not differ in either the fasted (n = 17/group) or fed (n = 5/group) state.

A number of studies have shown that increases in intracellular lipid metabolites can directly cause insulin resistance in both muscle and liver [39], [40]. Because iNKTs are lipid-sensing cells that are highly enriched amongst intrahepatic lymphocytes [13], we hypothesized that the primary metabolic disturbance in mice lacking CD1d (and therefore iNKTs) would most likely be hepatic lipid metabolism, with insulin resistance as a secondary consequence. We observed an increase in hepatic triglyceride accumulation with high fat feeding that was more severe in *CD1d^−/−^* mice (Figure 6A&B). Liver triglyceride accumulation appeared to be specific, as we did not detect any alterations in plasma triglycerides (Figure 6D), consistent with previously published work showing that CD1d deficiency did not alter plasma triglyceride or cholesterol content [14], [15], [16], [17], nor did we observe any difference in plasma free fatty acids in either the fed or fasted state (Figure 6E). Interestingly, other lipid classes were differently dysregulated, as there was a trend toward lower cholesterol content (Figure 6C). Because CD1d is known to bind various phospholipid species [41], [42], [43], we also analyzed liver lipids from obese *CD1d^−/−^* and WT mice by ^31^P NMR. Interestingly, several species appeared to be altered in both quantity and chemical shift (Figure S1 and Table 2). We noted that the two spectra could not be perfectly aligned, suggesting that the chemical composition of some of the major species was different between knockout and WT livers (Figure S1). When we manually assigned arbitrary identifiers (a–h) to individual peaks, we found that not only the species composition, but also the molarity were altered by genotype (Table 2). Thus liver lipid content is altered qualitatively and quantitatively in *CD1d^−/−^* mice when compared to wild type animals on high fat diet.

**Table 2:** Altered phospholipid composition of *CD1d^−/−^* livers.

	Species concentration (mM)							
	a	b	c	d	e	f	g	h
genotype	n/a	LPA	n/a	n/a	PA	n/a	n/a	n/a
**WT**	0.170	3.553	0.176	0.068	0.837	0.248	0.71	0.336
**KO**	0.176	4.246	0.231	0.116	0.928	0.398	0.169	0.161
**fold**	1.035	1.195	1.313	0.586	1.109	1.605	0.238	0.479

Phospholipid composition measured by ^31^P NMR shows that the quantity of several phospholipid species is altered in livers of obese *CD1d^−/−^* mice relative to controls (as demonstrated by fold changes). Most peaks could not be assigned, as indicated by “n/a” (analyzed from a single pooled sample of 8/group). LPA = lysophosphatidic acid; PA = phosphatidic acid.

**Figure 7:**
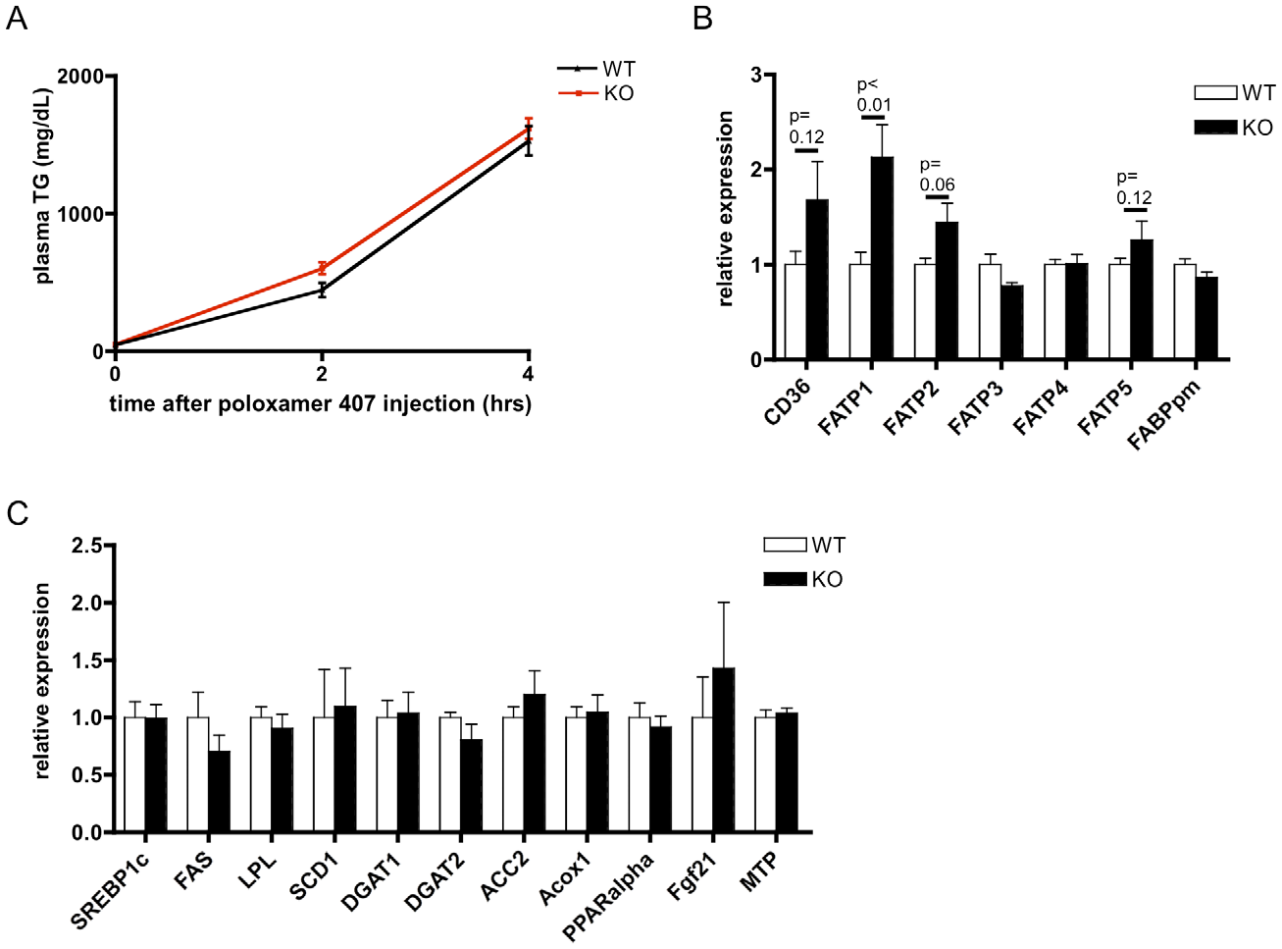
Steatosis in *CD1d^−/−^* mice is most likely due to increased fatty acid uptake. A) Hepatic triglyceride export (VLDL) is not decreased in DIO *CD1d^−/−^* mice (n = 10–11/group). B) qRT-PCR analysis of obese mouse livers shows increased expression of fatty acid transporters, but C) unchanged expression of fatty acid and triglyceride synthetic and oxidative genes (n = 8/group).

Hepatic steatosis occurs whenever the positive and negative fluxes of triglycerides in the liver are unequal. This could be due to an increase in *de novo* lipogenesis in the liver, an increase in lipid uptake from the plasma, a decrease in triglyceride export, or a reduction in fat oxidation in the liver. To explore the possibility of defective hepatic triglyceride export in high fat-fed *CD1d^−/−^* mice, we injected mice with poloxamer 407, a non-ionic surfactant that inhibits lipoprotein lipase and low-density lipoprotein receptor-mediated uptake of triglyceride into tissue such that triglyceride exported from the liver accumulates in plasma [44]. We found that hepatic export was not diminished in *CD1d^−/−^* mice (Figure 7A). To investigate other possible mechanisms indirectly, we examined gene expression of fatty acid and triglyceride metabolic genes in the liver. We found that *CD1d^−/−^* mice had no difference in expression of fatty acid synthetic or oxidative genes (Figure 7C), but had slightly higher expression of some fatty acid transporters (Figure 7B), suggesting increased lipid uptake as a potential mechanism underlying lipid accumulation in *CD1d^−/−^* livers. Consistent with a minor reduction of cholesterol content in *CD1d^−/−^* livers, several genes involved in sterol metabolism and detoxification were slightly altered in *CD1d^−/−^* livers (Figure S2).

### CD1d deficiency worsens metabolic parameters when mice are fed a choline deficient diet

**Figure 8:**
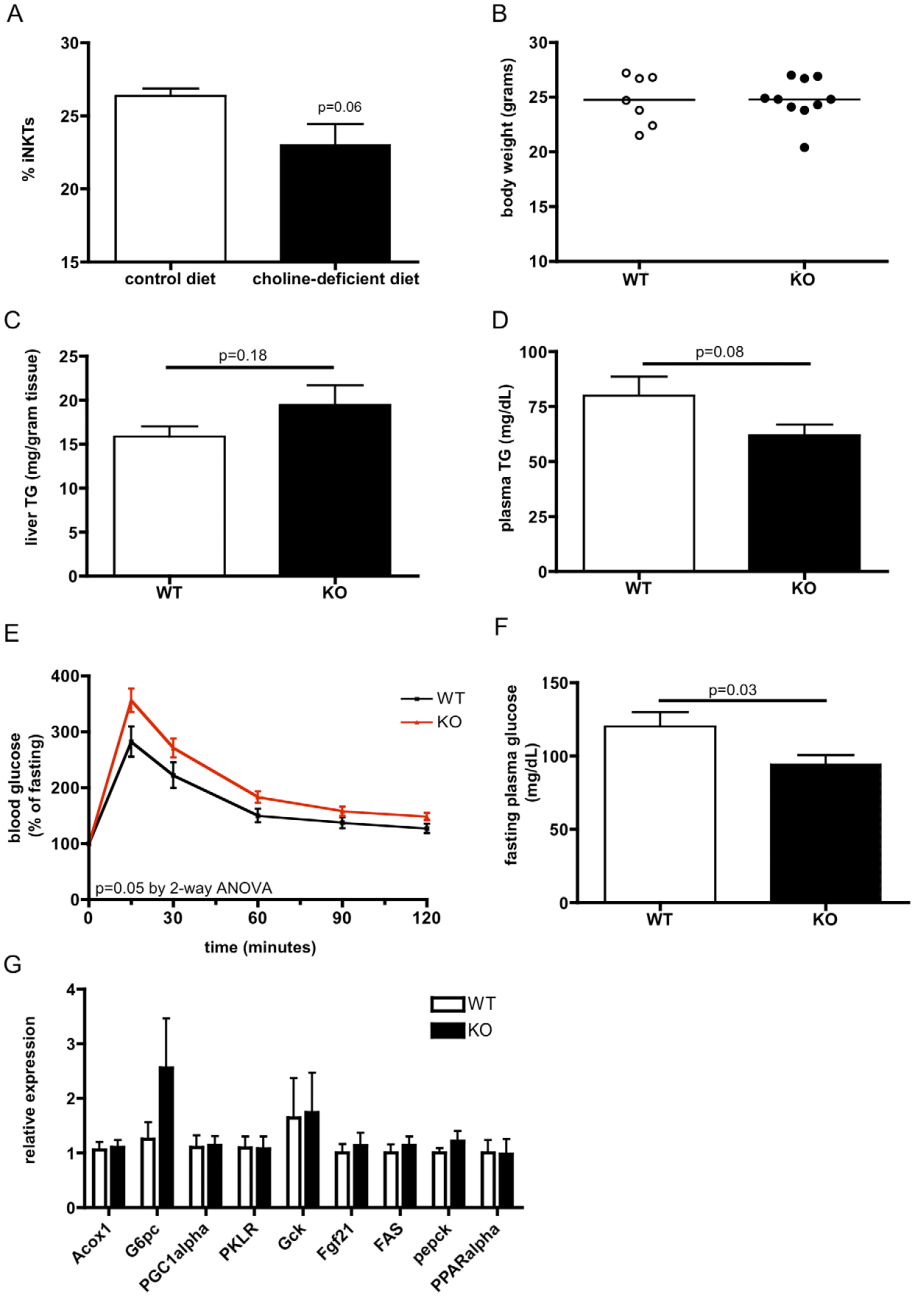
CD1d deficiency worsens metabolic parameters when mice are fed a choline-deficient diet. A) Hepatic iNKTs (as a % of B220- hepatic mononuclear cells) are (nonsignificantly) reduced by choline-deficient diet. B) Body weight is normal in both *CD1d^−/−^* and WT mice fed a choline-deficient diet. C) Liver triglycerides are slightly increased in *CD1d^−/−^* on choline-deficient diet, whereas D) plasma triglycerides are slightly decreased. E) Abnormal glucose tolerance observed in *CD1d^−/−^* on choline-deficient diet with F) decreased fasting plasma glucose. G) No differences in gene expression are observed in *CD1d^−/−^* livers (n = 7–10/group for all experiments).

To examine the effect on glucose homeostasis and hepatic lipid metabolism in another model of steatosis, independent of obesity, we examined *CD1d^−/−^* and WT mice fed a choline-deficient diet. As expected, choline-deficient diet feeding trended to decrease the proportion of hepatic iNKTs (Figure 8A), consistent with published data [45], although mice of both genotypes had a normal body weight (Figure 8B). Choline-deficient diet induced a non-significant increase in liver triglyceride content (Figure 8C) and a slight decrease in plasma triglyceride in *CD1d^−/−^* mice relative to controls (Figure 8D). Choline-deficient diet-fed *CD1d^−/−^*mice also showed an increased glucose excursion during a glucose tolerance test (Figure 8E), with decreased fasting plasma glucose (Figure 8F). No clear differences in gene expression that could explain the increased steatosis or altered glucose homeostasis were observed (Figure 8G). Thus, the overall metabolic profile of choline-induced steatosis resembled that induced by high-fat diet in *CD1d^−/−^* mice.

### iNKT deficiency does not alter fasting tolerance

**Figure 9:**
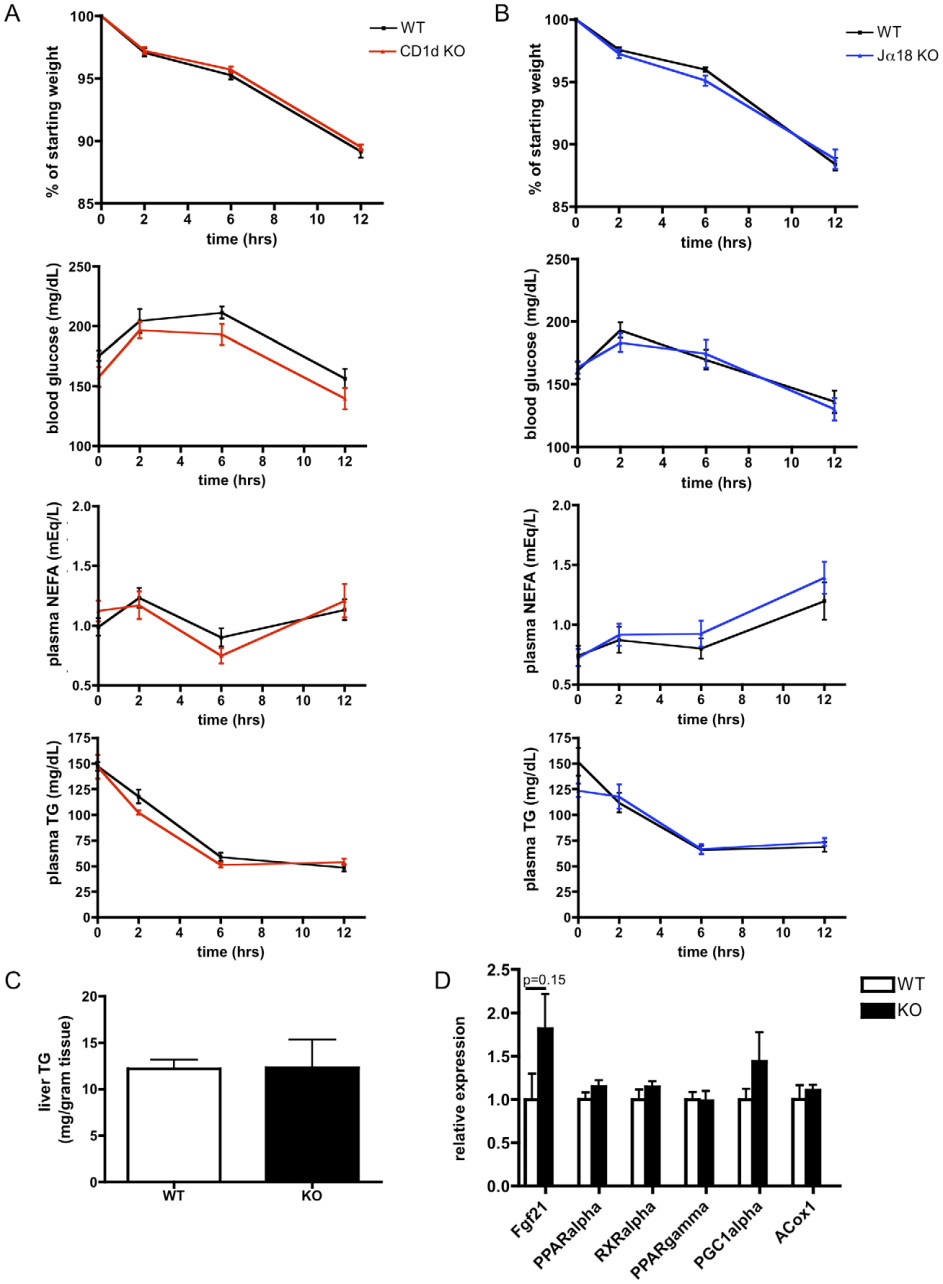
iNKT deficiency does not alter fasting tolerance. A) When fasted for 12 hours, *CD1d^−/−^* and WT mice lose an equal percentage of body weight, maintain similar blood glucose and FFAs, and experience a similar decay in plasma TG (n = 7–8/group). B) When fasted for 12 hours, *Jα18^−/−^* and WT mice lose an equal percentage of body weight, maintain similar blood glucose and FFAs, and experience a similar decay in plasma TG (n = 7–8/group). C) Triglyceride content does not differ between *CD1d^−/−^* and WT mice after fasting (n = 4/group). D) Minimal gene expression differences observed between fasted *CD1d^−/−^* and WT mice (n = 4/group).

Given the extremely modest effect of CD1d deficiency on glucose tolerance and hepatic steatosis during high fat feeding, we hypothesized that iNKTs may be more important for other types of metabolic challenges, such as fasting. During fasting, adipose tissue lipolysis mobilizes fatty acids and glycerol, which must be converted to ketones and glucose, respectively for use by other tissues. When *CD1d^−/−^* mice or controls were subjected to a 12-hr fast, they lost equal amounts of weight, maintained plasma glucose and free fatty acids at equivalent levels, and demonstrated an equal decrement in plasma triglycerides (Figure 9A). Plasma analytes were also similar in a separate experiment that extended the period of fasting to 24 hours (data not shown). Similarly, *Jα18^−/−^* mice, which lack iNKTs due to a disruption in the canonical Jα18-Vα14 TCR-alpha chain rearrangement [46] but are sufficient in CD1d, did not differ from controls in any of these parameters (Figure 9B). At the end of the fasting period, liver triglyceride levels between *CD1d^−/−^* mice and controls did not differ (Figure 9C). Gene expression of important oxidative genes induced during fasting did not differ between *CD1d^−/−^* mice and littermates (Figure 9D). These data suggest no obvious role for CD1d or iNKT cells in metabolic adaptation to fasting state.

### Major metabolic changes induced by LPS stimulation are not CD1d-dependent

**Figure 10:**
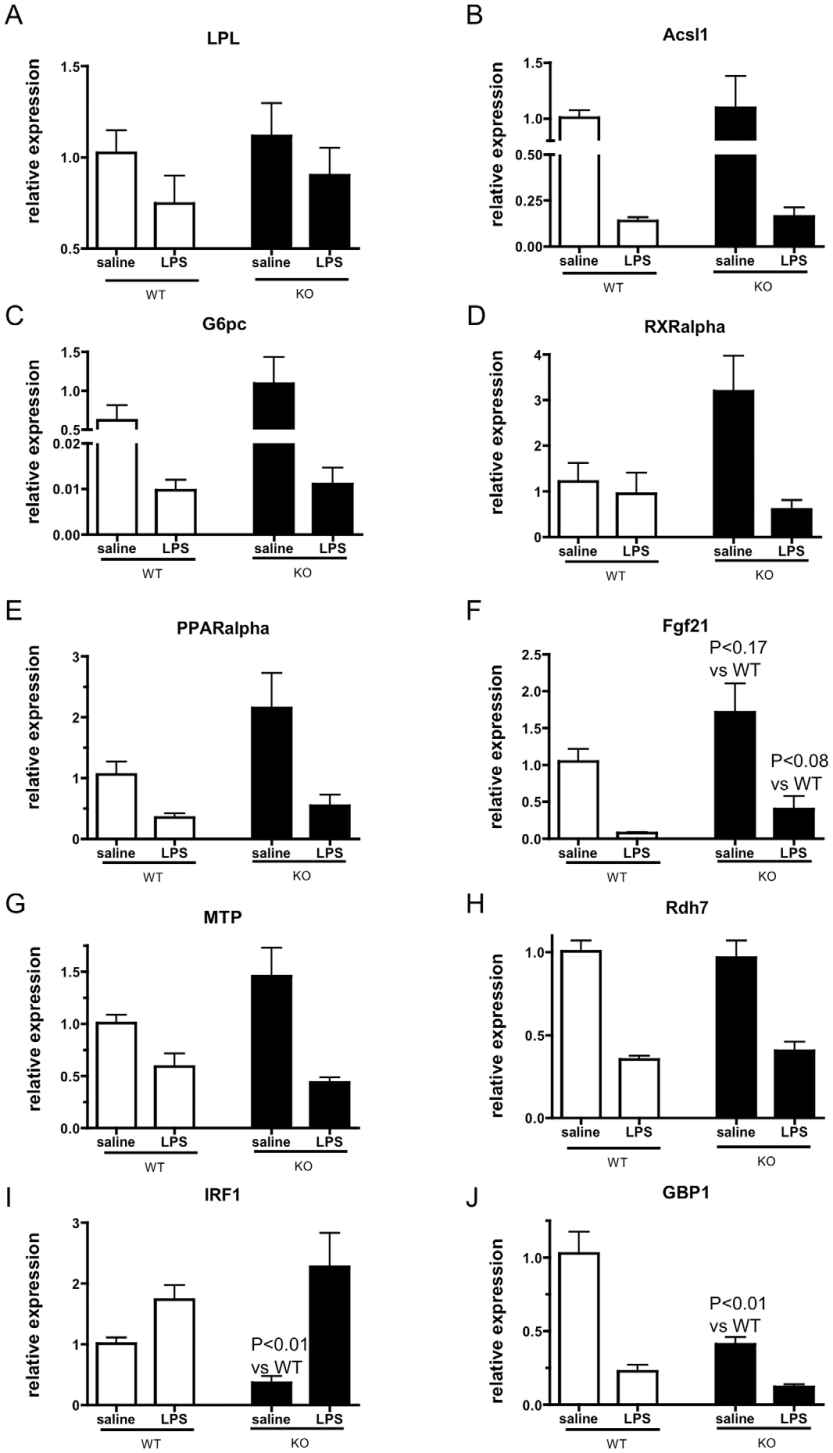
Major metabolic changes induced by LPS stimulation are not CD1d-dependent. A–H) Many genes altered by LPS stimulation are not affected by CD1d deficiency. I) *IRF1* expression is reduced in *CD1d^−/−^* mice at baseline but not after LPS stimulation. J) *GBP1* expression is reduced in *CD1d^−/−^* mice (n = 4/group for all).

Inflammation and infection are also important metabolic challenges for which iNKTs may be required. To test the role of CD1d in this model, we simulated infection by injecting *CD1d^−/−^* mice and controls with LPS and measured changes in metabolic gene expression. Among candidate metabolic genes previously shown to be changed in the liver by LPS stimulation [47], none were CD1d-dependent (Figure 10 A–H). Of interest, however, *Fgf21* trended to increase in both unstimulated (p = 0.17) and LPS-stimulated (p = 0.08) *CD1d^−/−^* livers (Figure 10F), as in fasted *CD1d^−/−^* livers (Figure 9D), suggesting there may be a subtle alteration of oxidation or substrate switch in *CD1d^−/−^* livers that could not be adequately revealed by any of our experiments. In comparison to metabolic genes, two interferon-responsive genes, *IRF1* and *GBP1*, had lower expression in *CD1d^−/−^* mice at baseline (Figure 10I&J), consistent with production of IFNγ by iNKTs in basal conditions [48]. We also found that *CD1d^−/−^* mice had almost no detectable *IL-4* mRNA in the liver (Figure S3) Although it is not known whether iNKT-derived IL-4, like IFNγ, is translated in the basal state, this may suggest that iNKTs are also important contributors to hepatic IL-4 production under basal conditions. Despite this difference, we did not observe differences in macrophage number or M2 macrophage gene expression. Thus, while iNKTs may contribute a significant portion of interferons α/β or γ and IL-4 in the liver in unchallenged conditions, these do not appear to contribute significantly to metabolic adaptations to acute LPS challenge in the liver.

### The metabolic phenotype of *CD1d^−/−^* mice is independent of iNKTs

**Figure 11:**
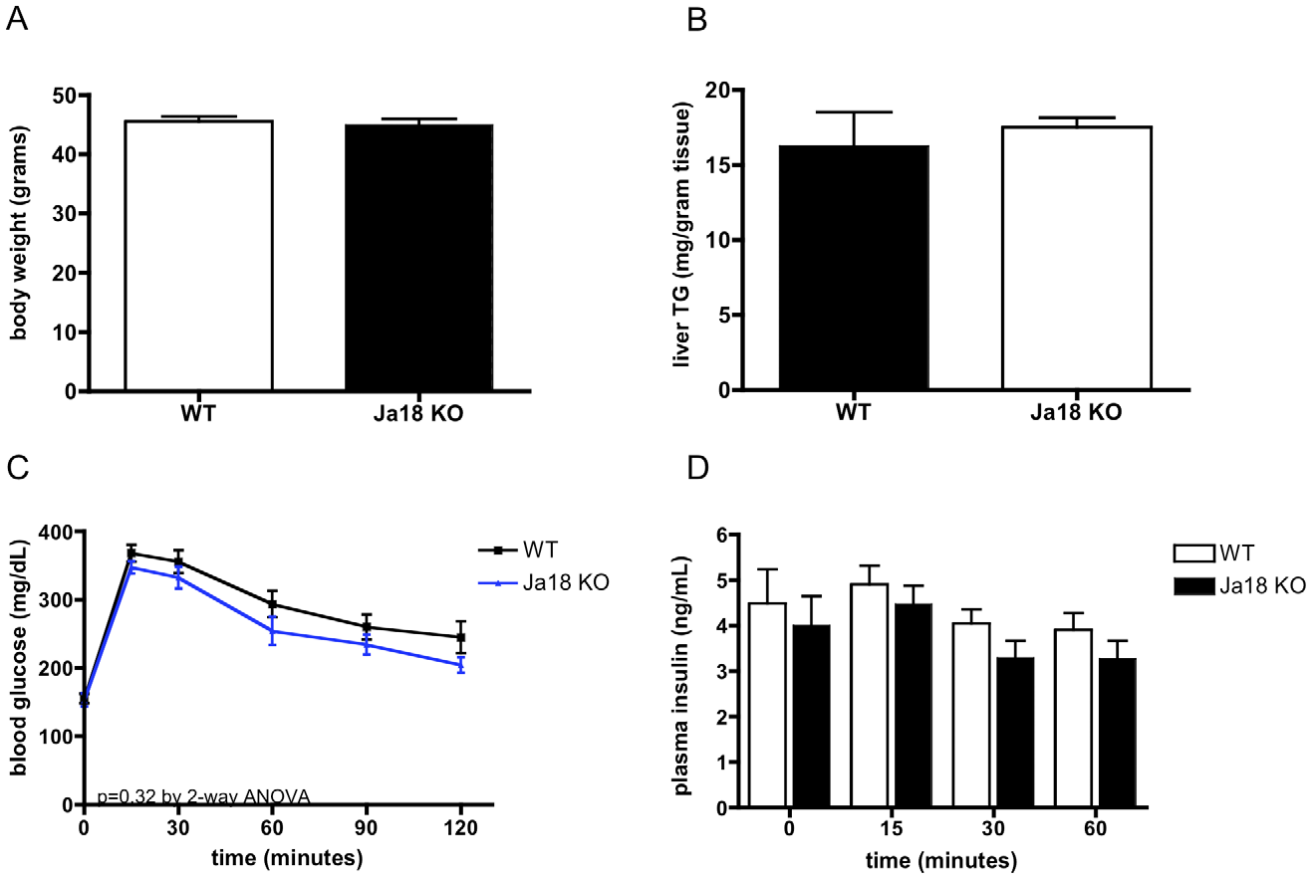
The metabolic phenotype of *CD1d^−/−^* mice is independent of iNKTs. A) Body weight does not differ between high fat-fed *Jα18^−/−^* and WT mice. B) Liver triglyceride is not different in high fat fed *Jα18^−/−^* and WT mice. C) Glucose tolerance is not different in high fat fed *Jα18^−/−^* and WT mice. D) Plasma insulin does not differ during a glucose tolerance test (n = 9–10/group for all panels).

*CD1d^−/−^* mice are deficient not only in iNKTs, but also other CD1d-restricted T cells with variable or semi-invariant T cell receptors [49], as well as CD1d itself. To verify that the phenotype we observed was attributable to iNKTs, we also examined *Jα18^−/−^* mice. Like *CD1d^−/−^* mice, *Jα18^−/−^* mice had identical body weight to WT mice on high fat diet (Figure 11A) After verifying by FACS that both strains were, indeed, deficient in iNKTs, we were surprised to find that high fat fed *Jα18^−/−^* mice did not develop increased hepatic steatosis (Figure 11B) Moreover, *Jα18^−/−^* mice, unlike *CD1d^−/−^* mice, did not show any trend towards glucose intolerance on a glucose tolerance test (Figure 11C), nor did they exhibit abnormally elevated plasma insulin during the glucose tolerance test (Figure 11D). These data suggest that the small differences we observed in hepatic triglyceride accumulation and insulin resistance in *CD1d^−/−^* mice are independent of iNKTs and are instead dependent on CD1d-restricted non-iNKT cells, or on a T-cell-independent role of CD1d. Additionally, although both strains have been backcrossed to C57Bl/6 at least 6 times, we cannot rule out the possibility that the phenotypic differences seen are attributable to small genetic differences other than *CD1d* or *Jα18*, including linked genes.

## Discussion

Previous investigators have noted decreases in hepatic iNKTs during obesity and have implicated iNKTs in either helpful or pathologic roles in metabolic syndrome [14], [15], [16], [17], [18], [19], [20], [21], [22], [23]. However, these studies have not examined hepatic lipid content or whole body glucose homeostasis in *CD1d^−/−^* mice or *Jα18^−/−^* mice. Data utilizing knockout or depletion of multiple cell types simultaneously are difficult to interpret in light of data demonstrating a role for other types of T cells in metabolism [4], [5], [6]. However, during the preparation of this manuscript, a study was published in which high fat-fed *CD1d^−/−^* mice were reported to have glucose tolerance indistinguishable from wild type and a non-significant trend to increased hepatic triglyceride accumulation [32]. These data are consistent with ours, and may differ only in the exceptionally large numbers of animals required to resolve the small differences we observed, as discussed further below. Furthermore, our data suggest that the subtle differences observed between *CD1d^−/−^* mice and their littermates are not attributable to iNKTs, as *Jα18^−/−^* mice do not recapitulate the phenotype. Thus, we believe that iNKTs play a minimal if any role in metabolism in the context of diet-induced obesity, while CD1d plays a small but potentially important role in obesity. However, we cannot rule out the possibility that iNKTs and/or CD1d play an important role in metabolic control in a context not recapitulated by our experiments.

Like other investigators before us [19], [20], [21], we observed a selective decrease in iNKTs in the liver of obese animals that was not seen in the spleen or white adipose, consistent with previously published results [4], [5], [6], [32]. We did not detect any defect in proliferation or maturation of these cells, nor evidence of acute activation. Other investigators have suggested that increased apoptosis, perhaps due to lipotoxicity, is the cause of reduced hepatic iNKTs [20]. However, we continue to consider the possibility that iNKTs may be depleted from fatty livers because of activation or exhaustion-induced cell death, rather than non-specific lipotoxicity. A final possible cause of hepatic iNKT depletion is decreased CD1d and CXCL16-dependent retention in the liver, perhaps combined with potential upregulation of these signals in distant sites.

Although indistinguishable from littermates on a chow diet, *CD1d^−/−^* mice develop slightly exacerbated insulin resistance on a high fat diet. Importantly, a large number of animals were needed to resolve differences seen on a GTT, indicating that the effect size conferred by CD1d genotype is quite small when compared against stochastic inter-animal variation seen during this test. When a perfectly weight-matched group of mice was analyzed using hyperinsulinemic-euglycemic clamps, a significant worsening of hepatic insulin resistance was observed in obese *CD1d^−/−^* mice, while no significant change in peripheral glucose uptake was observed. This suggested that the mild exacerbation of insulin resistance in *CD1d^−/−^* mice was due to hepatic insulin resistance.

Accompanying glucose intolerance in both the high fat-fed and choline-deficient diet-fed mice was increased hepatic TG content. Phospholipid composition also appeared to be altered, with both peak amplitude and chemical shift differing between obese *CD1d^−/−^* mice and controls. Such chemical shifts may suggest altered acyl chain length or level of saturation. More elusive, however, is the cause of these lipid changes. We observed no difference in hepatic triglyceride export, gene expression related to *de novo* triglyceride synthesis, or gene expression related to fat oxidation. However, we did observe increased hepatic expression of several fatty acid transporters, suggesting that increased lipid flux into the liver could be the proximal mechanism leading to steatosis in these mice. Preference of these transporters for specific acyl chain types could underlie the altered hepatic phospholipid composition. Also interesting is the possibility that CD1d itself may play some role in transport of lipids, perhaps contributing qualitatively or quantitatively to the lipid composition changes we observed.

Because the metabolic changes observed in high fat-fed *CD1d^−/−^* mice were very small in magnitude, we hypothesized that high fat feeding was not the most relevant challenge and that iNKTs and/or CD1d might be more important for metabolic adaptation during different types of challenges. Other than obesity, fasting and inflammation are two states in which metabolic demands are dramatically changed. When we examined *CD1d^−/−^* mice or *Jα18^−/−^* mice during fasting, however, we observed no difference in any measured metabolic parameter compared to controls. Similarly, in LPS-challenged mice, gene expression changes in metabolic pathways were not altered by CD1d expression. A small trend towards increased *Fgf21* expression was noted, however, in fasted mice, *ad libitum* fed mice, and *ad libitum* fed LPS-injected mice. Interestingly, recent data from Ricardo-Gonzalez et al. suggested that IL-4 signaling through STAT-6 in the liver could inhibit expression of PPARα target genes such as *Fgf21* [50]. Given the dramatic reduction in *IL-4* expression in *CD1d^−/−^* livers, this may suggest that iNKTs contribute some but not all of the IL-4 protein in the liver under basal conditions, leading to a mild version of the phenotype observed by Ricardo-Gonzalez et al. This phenotype does not appear to be connected to either the steatosis or glucose intolerance observed in our model, as these were iNKT-independent. While it is well known that iNKTs express many untranslated cytokine mRNAs in the unstimulated state [51], and that some translation of IFNγ mRNA occurs in normal, healthy mice [48], it is not yet clear whether IL-4 is also translated. Our observed changes in *Fgf21* expression may suggest that IL-4 translation does occur, but does not appear to contribute substantially to metabolic control in any of the experimental paradigms examined in this study.

IL-4 is also thought to be important for polarizing macrophages towards an “alternatively activated” state. Alternatively activated macrophages, in turn, have been suggested to improve oxidative metabolism, counteracting the insulin resistance induced by “classically activated” inflammatory macrophages [52], [53], [54]. Despite the reduction in IL-4 expression in *CD1d^−/−^* mice, we did not observe any difference in macrophage numbers or polarization. Therefore, iNKT-derived cytokines are unlikely to be responsible for the observed metabolic changes in *CD1d^−/−^* mice.

Some data presented herein may suggest that the metabolic phenotype of *CD1d^−/−^* mice is completely independent of T cells: CD1d expression is regulated in the brain, for example, which is devoid of T cells under normal conditions. Few roles for CD1d outside of antigen presentation to T cells have been elucidated, although some investigators have suggested that CD1d could signal internally to induce NKT-independent cytokine production [55]. However, because CD1d can bind and shield hydrophobic acyl chains and traffic between cellular compartments, it is possible to imagine its involvement in lipid transport outside of antigen presentation or cytokine production. For example, while microsomal triglyceride transfer protein (MTP) assists in CD1d lipidation, CD1d might contribute amphipathic lipids for MTP-dependent VLDL assembly. It will be very interesting to see whether CD1d does indeed play such a role, and whether this contributes to the altered lipid composition of *CD1d^−/−^* livers.

In conclusion, we have found that *CD1d^−/−^* mice have mildly exacerbated hepatic triglyceride accumulation associated with hepatic insulin resistance when fed a steatogenic diet. There is ample circumstantial evidence to suggest that iNKTs would be appropriate mediators of lipid metabolism in the liver, as well as evidence that the absence of iNKTs led to reductions in cytokines shown to be important in metabolism. Surprisingly, however, the phenotypes we observed in *CD1d^−/−^* mice did not repeat in *Jα18^−/−^* mice, and thus are likely to be independent of iNKTs. While we were unable to find any role for CD1d or iNKTs in inflammation or fasting-induced metabolic changes, and only a minimal role for CD1d in the context of high fat feeding, it remains possible that CD1d and/or iNKTs play an important role in metabolic control in a context not recapitulated by our experiments, or in general, outside of the natural environment.

## Supporting Information

Figure S1 **Altered phospholipid composition of** ***CD1d^−/−^*** **livers.** Phospholipid composition measured by ^31^P NMR shows that the quantity and chemical shift of several phospholipid species is altered in livers of obese *CD1d^−/−^* mice relative to controls (analyzed from a single pooled sample of 8 mice/group).
(TIF)

Figure S2 **Altered expression of genes involved in sterol metabolism and detoxification in** ***CD1d^−/−^*** **livers.** (n = 8 mice/group).
(TIF)

Figure S3 **Macrophage-related gene expression in** ***CD1d^−/−^*** **mice.** (n = 8 mice/group).
(TIF)

Table S1 **Gene list for quantitative PCR analyses.**
(TIF)
