# Supplementary figures and images for: Impact of CD1d Deficiency on Metabolism
Source: PLoS One. 2011 Sep 29;6(9):e25478. doi: 10.1371/journal.pone.0025478 (PMC3183002; doi:10.1371/journal.pone.0025478)

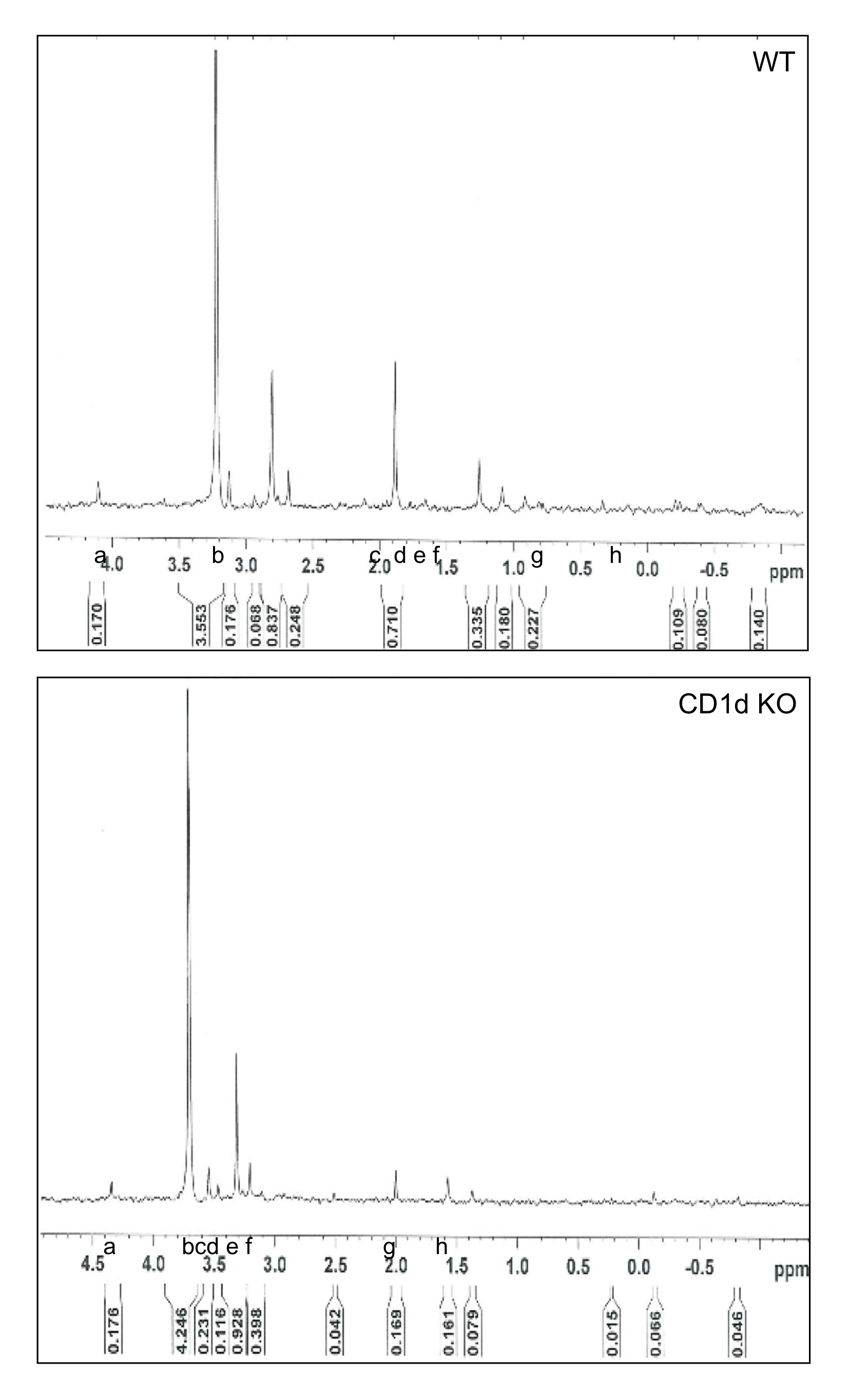

Supplement: Figure S1 — Altered phospholipid composition of CD1d−/− livers. Phospholipid composition measured by 31P NMR shows that the quantity and chemical shift of several phospholipid species is altered in livers of obese CD1d−/− mice relative to controls (analyzed from a single pooled sample of 8 mice/group). (TIF) [file pone.0025478.s001.tif]

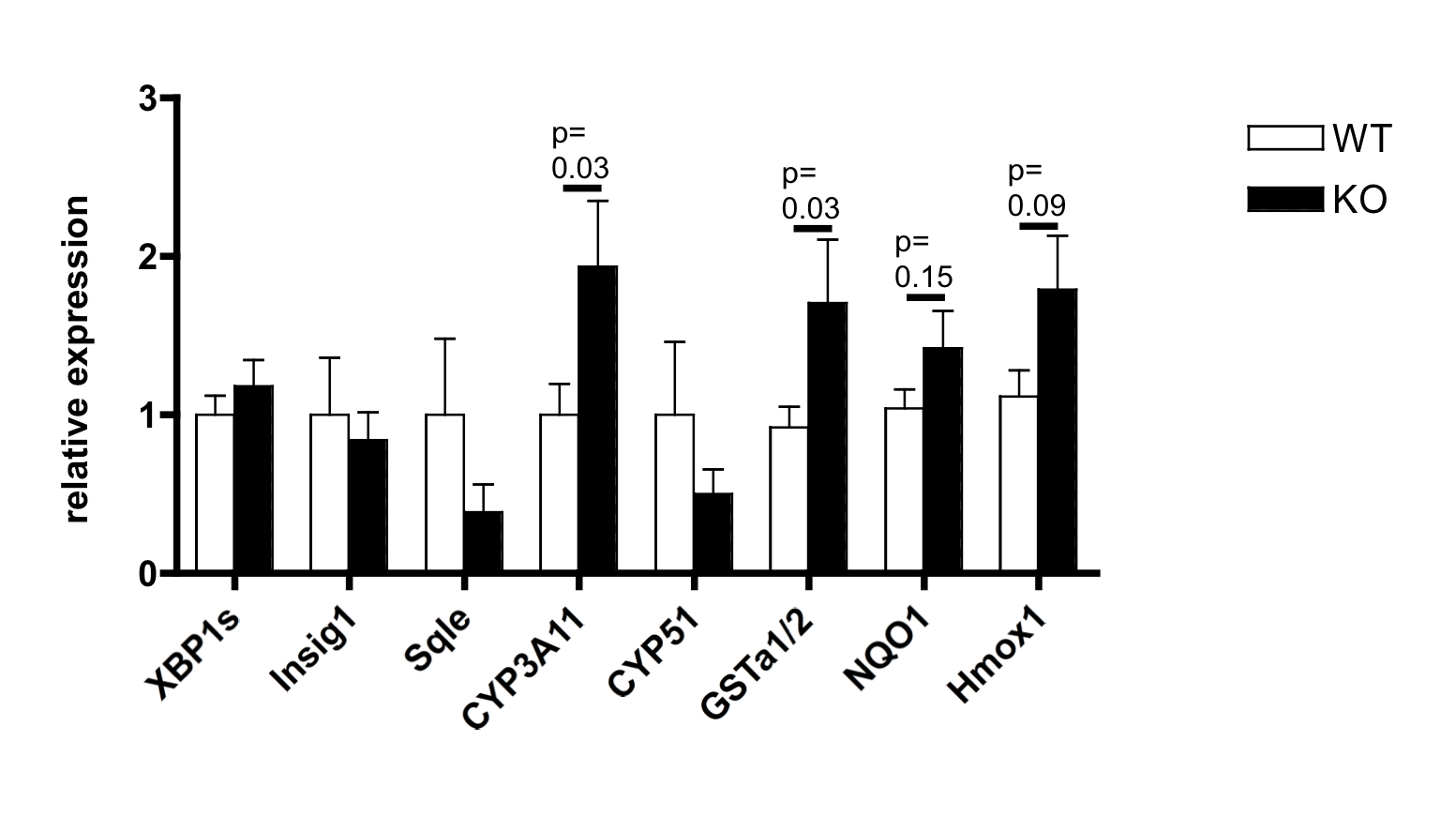

Supplement: Figure S2 — Altered expression of genes involved in sterol metabolism and detoxification in CD1d−/− livers. (n = 8 mice/group). (TIF) [file pone.0025478.s002.tif]

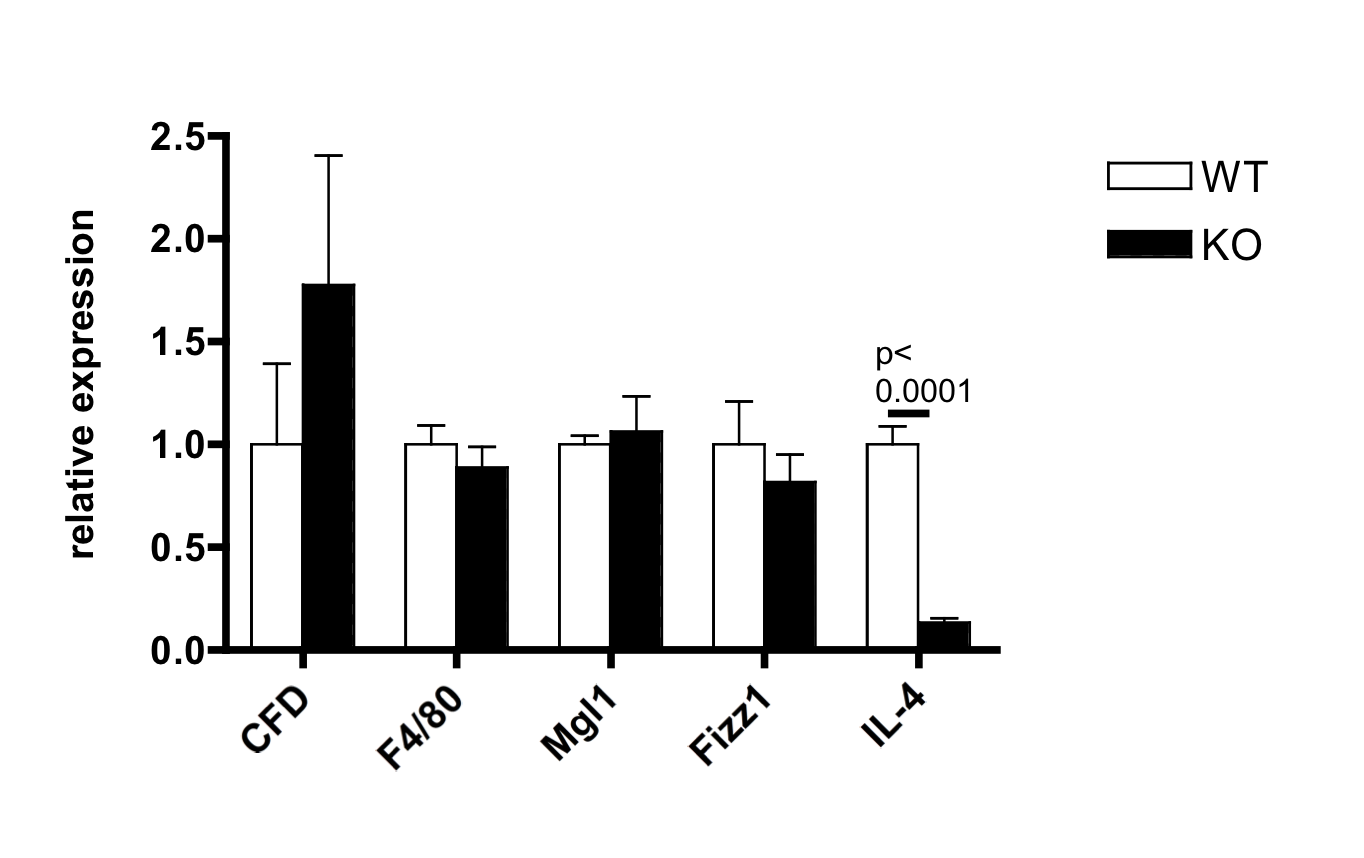

Supplement: Figure S3 — Macrophage-related gene expression in CD1d−/− mice. (n = 8 mice/group). (TIF) [file pone.0025478.s003.tif]

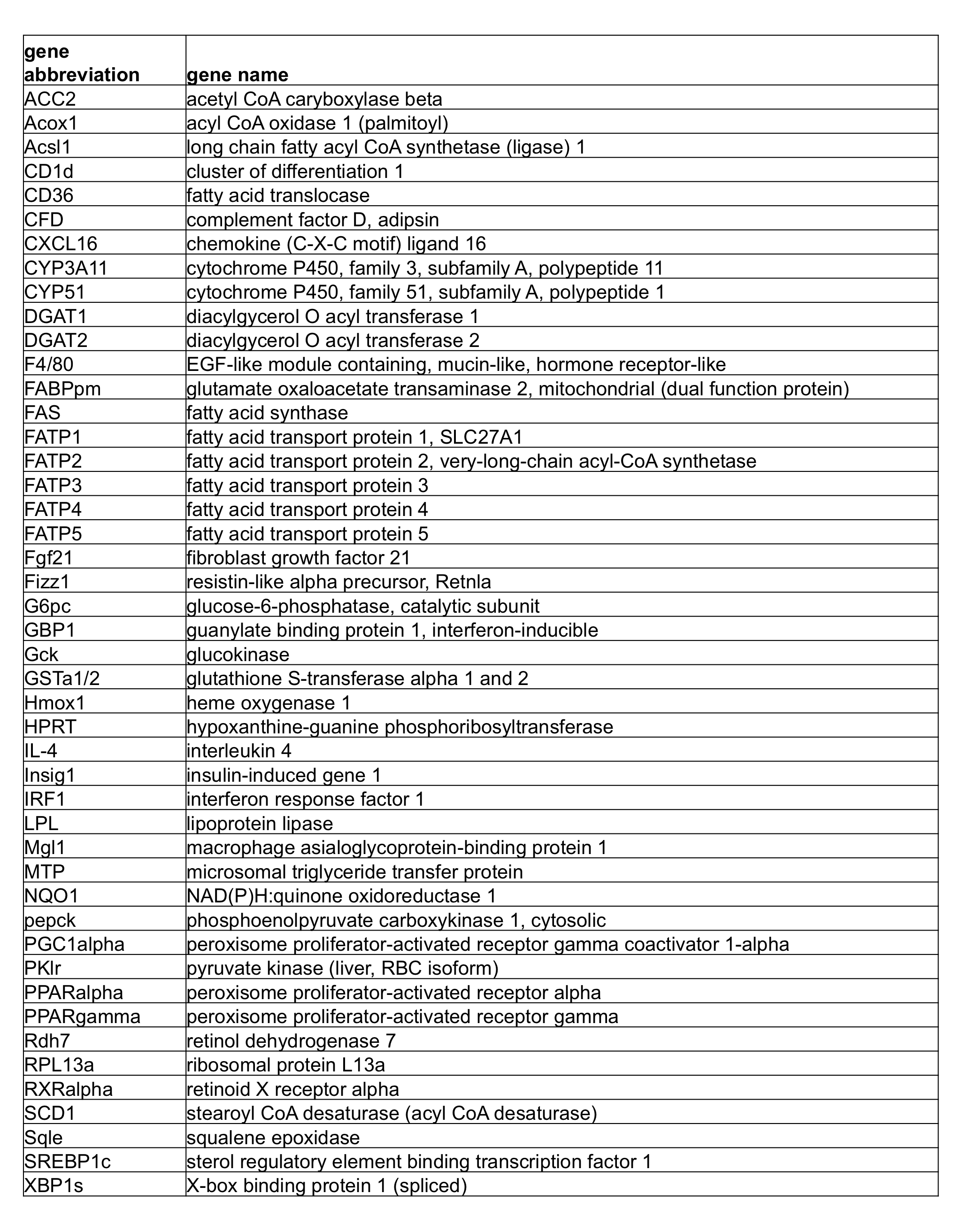

Supplement: Table S1 — Gene list for quantitative PCR analyses. (TIF) [file pone.0025478.s004.tif]
